# Haplotype analysis suggest that the *MLH1* c.2059C > T mutation is a Swedish founder mutation

**DOI:** 10.1007/s10689-017-0067-x

**Published:** 2017-12-29

**Authors:** Jenny von Salomé, Tao Liu, Markku Keihäs, Moni Morak, Elke Holinski-Feder, Ian R. Berry, Jukka S. Moilanen, Stéphanie Baert-Desurmont, Annika Lindblom, Kristina Lagerstedt-Robinson

**Affiliations:** 1 Department of Molecular Medicine and Surgery, Karolinska Institutet, and Clinical Genetics, Karolinska University Hospital, Solna, Stockholm, Sweden; 2 Department of Molecular Medicine and Surgery, Karolinska Institutet, Stockholm, Sweden; 3 Medizinische Klinik und Poliklinik IV, Campus Innenstadt, Klinikum der Universität München, Munich, Germany; 4 MGZ – Medizinisch Genetisches Zentrum, Munich, Germany; 5 Leeds Genetics Laboratory, St James’s University Hospital, Leeds, UK; 6 PEDEGO Research Unit and Medical Research Center Oulu, University of Oulu, Oulu, Finland; 7 Department of Clinical Genetics, Oulu University Hospital, Oulu, Finland; 8 Department of Genetics, Rouen University Hospital, Normandy Centre for Genomic and Personalized Medicine, Inserm U1079, IRIB, University of Rouen, Normandy University, Rouen, France

**Keywords:** Lynch syndrome, MLH1, Missense mutation, Founder mutation, Haplotype

## Abstract

Lynch syndrome (LS) predisposes to a spectrum of cancers and increases the lifetime risk of developing colorectal- or endometrial cancer to over 50%. Lynch syndrome is dominantly inherited and is caused by defects in DNA mismatch-repair genes *MLH1, MSH2, MSH6* or *PMS2*, with the vast majority detected in *MLH1* and *MSH2*. Recurrent LS-associated variants observed in apparently unrelated individuals, have either arisen de novo in different families due to mutation hotspots, or are inherited from a founder (a common ancestor) that lived several generations back. There are variants that recur in some populations while also acting as founders in other ethnic groups. Testing for founder mutations can facilitate molecular diagnosis of Lynch Syndrome more efficiently and more cost effective than screening for all possible mutations. Here we report a study of the missense mutation *MLH1* c.2059C > T (p.Arg687Trp), a potential founder mutation identified in eight Swedish families and one Finnish family with Swedish ancestors. Haplotype analysis confirmed that the Finnish and Swedish families shared a haplotype of between 0.9 and 2.8 Mb. While *MLH1* c.2059C > T exists worldwide, the Swedish haplotype was not found among mutation carriers from Germany or France, which indicates a common founder in the Swedish population. The geographic distribution of *MLH1* c.2059C > T in Sweden suggests a single, ancient mutational event in the northern part of Sweden.

## Introduction

Lynch syndrome (LS) is the most common hereditary colorectal cancer syndrome worldwide, representing 2–4% of the total colorectal cancer burden [1, 2]. Among individuals with a cancer diagnosis at young age the proportion is much higher. Patients have an increased risk for tumors primarily in the proximal colon and the lining of the endometrium, but also in sites such as the stomach, ovaries, small bowel, kidney, urinary tract and brain [3].

LS has an autosomal dominant pattern of inheritance and is caused by germline mutations in any of the DNA mismatch-repair genes *MLH1, MSH2, MSH6, PMS2* and *EPCAM* [4]. Because of incomplete penetrance and variable age of cancer development not all mutation carriers develop cancer. Still, there is up to 70% lifetime risk to come down with early onset colorectal- or endometrial cancer, with the characteristic accelerated development from adenoma to carcinoma [3]. To date just above 3000 sequence variants have been reported for *MLH1, MSH2, MSH6* and *PMS2* (http://insight-group.org/variants/database, accessed August 25th, 2016). The vast majority are described in *MLH1* and *MSH2*, accounting for approximately 76% of all mutations detected in Swedish Lynch families [5]. Families with a mutation within *MLH1* or *MSH2* commonly fulfil the Amsterdam I criteria [6] and have a mean age of CRC onset of between 43 and 46 years [7].

Most mutations in the MMR genes are family specific; still some mutations are observed in several different geographic or ethnic populations. Some of them recur in unrelated families because of sequence characteristics that make DNA prone to mutation [8]. Other mutations, so called founder mutations, arose at different occasions in single individuals and fanned out by succeeding generations and therefore show a high frequency in specific ethnic groups. Founder mutations are common in mendelian disorders and have been described in genetically isolated populations as well as in populations with a migratory history [9]. To date, at least 55 LS-associated founder mutations have been identified [8]. Examples are the *MLH1* exon 16 deletion and the *MLH1* substitution c.454-1G>A, two mutations that together account for up to 50% of LS in the eastern part of Finland [10]. Other examples are the *MSH2* c.1906G>C mutation, that accounts for about 20% of all cases of LS in Ashkenazi Jews [11], and the American founder in *MLH1* c.589-2A>G [12].

For several LS-associated founder mutations, a common origin has been verified on the basis of shared haplotypes. Moreover, the regional distribution of a mutation can suggest the origin of the mutation. In Sweden three MMR founder mutations have previously been reported; two substitutions in *MSH2* [13] and one frameshift mutation in *PMS2* [14]. In this study, we have analyzed the disease associated haplotype of the *MLH1* missense mutation c.2059C>T, detected in ten families of Swedish origin [5]. This mutation was first reported in Poland by Jakubowska in 2001 [15] and classified as pathogenic in 2013 [16], and has been detected in geographically diverse populations such as Japan, Australia, Germany, Spain and Italy. Our aim was to determine whether the Swedish LS families shared disease associated haplotype, and if so, investigate if this haplotype was present also in other families, from other countries, carrying the same mutation.

## Materials and methods

### Patients

Families from Stockholm County, Sweden, with suspected LS were referred to the Department of Clinical Genetics at the Karolinska University Hospital in Stockholm. After genetic counseling, suspected mutation carriers voluntarily took part in genetic screening of the MMR genes, performed as described [17]. Ten families were found to carry the *MLH1* c.2059C>T variant in Sweden [5]. Eight of those were identified at the Karolinska University Hospital in Stockholm between 1994 and 2015, and were consequently enrolled in this study (clinical data presented in Table 1). In addition, two mutation carriers (brothers) from Finland were included, as well as three families from Germany and one family from France. The Finnish family has Swedish ancestry, originating from an area close to the Swedish border in the northern part of Finland. Medical histories and pedigrees were collected from the Swedish families by direct interviews of probands or other family members. Tumor diagnoses were confirmed by pathology reports or hospital records, and age at cancer diagnoses were recorded for the individuals affected. Clinical information regarding the French and German families was available to some extent, while such information was missing for the Finnish family.

**Table 1:** Clinical features of the Swedish families carrying the *MLH1* c.2059C>T (p.Arg687Trp) mutation

Family	Generations in pedigree	Number of individuals in pedigree	Number of known carriers	Number of diagnosis in carriers	Number of carriers with diagnosis	Ages at diagnosis in carriers	Cancer in non-carrier/not tested patients (age present if known)
1552	5	18	4	2 CRC 1 EC	2 1	31–52 53	1 CRC at 40^d^
1894	4	30	5	2 CRC	2	63-^b^	3 CRC^d^
19	4	80	4	4 CRC 1 PC	4 1^a^	45–69 80	4 CRC^d^ 1 SC^c^ 1 liver ca^d^ 1 brain ca^d^ 1 oesophagus ca^d^ 1 kidney ca^c^
765	5	40	5*	3 CRC	3	38-^b^	
1197	5	56	13	10 CRC 1 EC 2 SC	9^a^ 1^a^ 1	41–80 78 71, 80	1 CRC^c^ 2 CRC^d^
1517	4	11	2	1 CRC 1 OV CA	1 1	48 53	1 CRC at 80 years^c^
2143	4	20	2	1 CRC	1	60	1 liver and lung ca^c^ 1 CA UNS at 49 years^d^
F0009520	2	2	2	2 CRC	2	57, 67	
10	3	15	1	1 CRC	1	49	1 CRC at 58^d^
11	n/a	n/a	1	1 CRC	1	36	n/a
12	n/a	n/a	1	1 CRC	1	37	n/a
13	n/a	n/a	1	1 CRC	1	37	n/a

*CRC* colorectal cancer, *EC* endometrial cancer, *PC* pancreas cancer, *OV CA* ovarian cancer, *SC* skin cancer, *CA UNS* unspecified tumor location, *ca* cancer

^a^Patient with two tumor diagnosis

^b^Unknown age

^c^Verified non-carrier

^d^Not tested

*One individual with both *PMS2* and *MLH1* mutation

### Patients and TaqMan analysis in prevalence study

The case cohort in the prevalence study was composed of 2982 consecutive CRC patients which were enrolled in a national study. Patients underwent surgery in Stockholm or Uppsala between 2004 and 2009. They were interviewed by the same person about their family history of colorectal cancer and other malignancies. Cancer in first- and second-degree relatives and cousins was recorded, as well as tumour location, sex and age of the index-patients based on the medical records. All tumours were evaluated directly after surgery by a local pathologist. The control cohort was composed of 1610 anonymous blood donors from the same geographic region as the CRC patients, including 448 spouses to the CRC patients who did not have cancer and no family history of cancer. Screening of the c.2059C>T mutation in the colon cancer cases and control group were performed using TaqMan SNP Genotyping Assay (Applied Biosystems, Foster City, CA) according to the manufacturer´s instructions.

### Haplotype analysis

Haplotype analysis was initially performed in one selected family (Family 1552) with more than three known mutation carriers spanning over more than two generations, in order to determine the shared haplotype carrying the disease associated allele. In this family, three individuals were genotyped. In four Swedish families two individuals were genotyped to verify the disease haplotype (families 1894, 765, 1197 and 2143). In the remaining three Swedish families (19, 1517 and F0009520) only the index case was available for genotyping. Two individuals (brothers) from a Finnish family carrying the *MLH1* c.2059C>T mutation (family 9) were genotyped. Regarding the French family (family 10) and the three German families (family 11–13) with this mutation, only the index case was available for genotyping.

Genomic DNA from the mutation carriers were initially analyzed using 19 polymorphic microsatellite markers surrounding the *MLH1* gene, located on chromosome 3p22.2 (D3S1263, D3S2338, D3S1266, D3S3518, D3S1619, D3S1612, D3D3512, D3S1277, D3S3718, D3S2411, D3S1561, D3S1611, D3S2417, D3S3623, D3S1298, D3S3939, D3S1260, D3S3521 and D3S1289). The markers were selected using the UCSC database (http://genome.ucsc.edu/), human assembly GRCh37. Markers were viewed using full view of STS (sequence-tagged site) markers in track “Mapping and sequencing”. Polymorphic markers, primarily markers in the deCode database were highlighted under STS markers track settings. Markers in the investigated region (11.5–54.5 Mb according to human assembly GRCh37) were selected. These markers span a genomic region of 43.0 Mb with the *MLH1* c.2059C>T (p.Arg687Trp) mutation (37.09 Mb) situated between the markers D3S1611 (37.07 Mb) and D3S2417 (37.43 Mb). When a common haplotype was found in the Swedish families, only the shared genomic region was further analyzed in the Finnish, French and three German families. Primers were pooled and amplified using Type-it Microsatellite PCR Kit according to the manufacturer’s instructions (QIAGEN, Hilden, Germany). PCR-products were analyzed using 3500xL Genetic Analyzer and GeneMapper v5 according to the manufacturer’s protocol (Applied Biosystems, Thermo Fisher Scientific, Waltham, MA, USA).

The local Ethics Committee at Karolinska Institutet has approved this study, which followed the tenets of the Declaration of Helsinki.

## Results

### Clinical data

Eight families carrying the *MLH1* c.2059C>T mutation were identified at the Karolinska University Hospital in Stockholm. All families are, to ours and to the families understanding, unrelated. Genetic counseling was sought due to suspicion of inherited cancer and the number of family members in each pedigree varied considerably, as well as the number of genetically tested individuals. The clinical data is summarized in Table 1. There were 29 colorectal cancers in 28 patients and two endometrial cancers in two patients. In addition, there was one patient with ovarian cancer. The first CRC was diagnosed at a median age of 58.5 years (mean 55.5 years, range 31–80 years) and the first EC was diagnosed at a median age of 65.5 years (mean 65.5 years, range 53–78 years). One woman with CRC also developed EC, in which the CRC preceded the EC by 18 years. Two men with CRC also had other malignancies, including prostate cancer in one patient and malignant melanoma in the other.

### Prevalence of MLH1 c.2059C>T

Among 1610 normal controls, none were carriers of the mutation. Within the cohort of CRC cases, only one individual was a carrier of the mutation (1/2983) and this family (Family 2143) was included in our study. The index patient was first diagnosed with CRC cancer when she was 60 and had turned 72 by the time of this study. Her sister passed away at an age of 69 suffering from lung and liver cancer (Table 1). Only the index patient and one of her two daughters were genetically tested and proven to be mutation carriers.

### Outcome of haplotype analysis

**Fig. 1:**
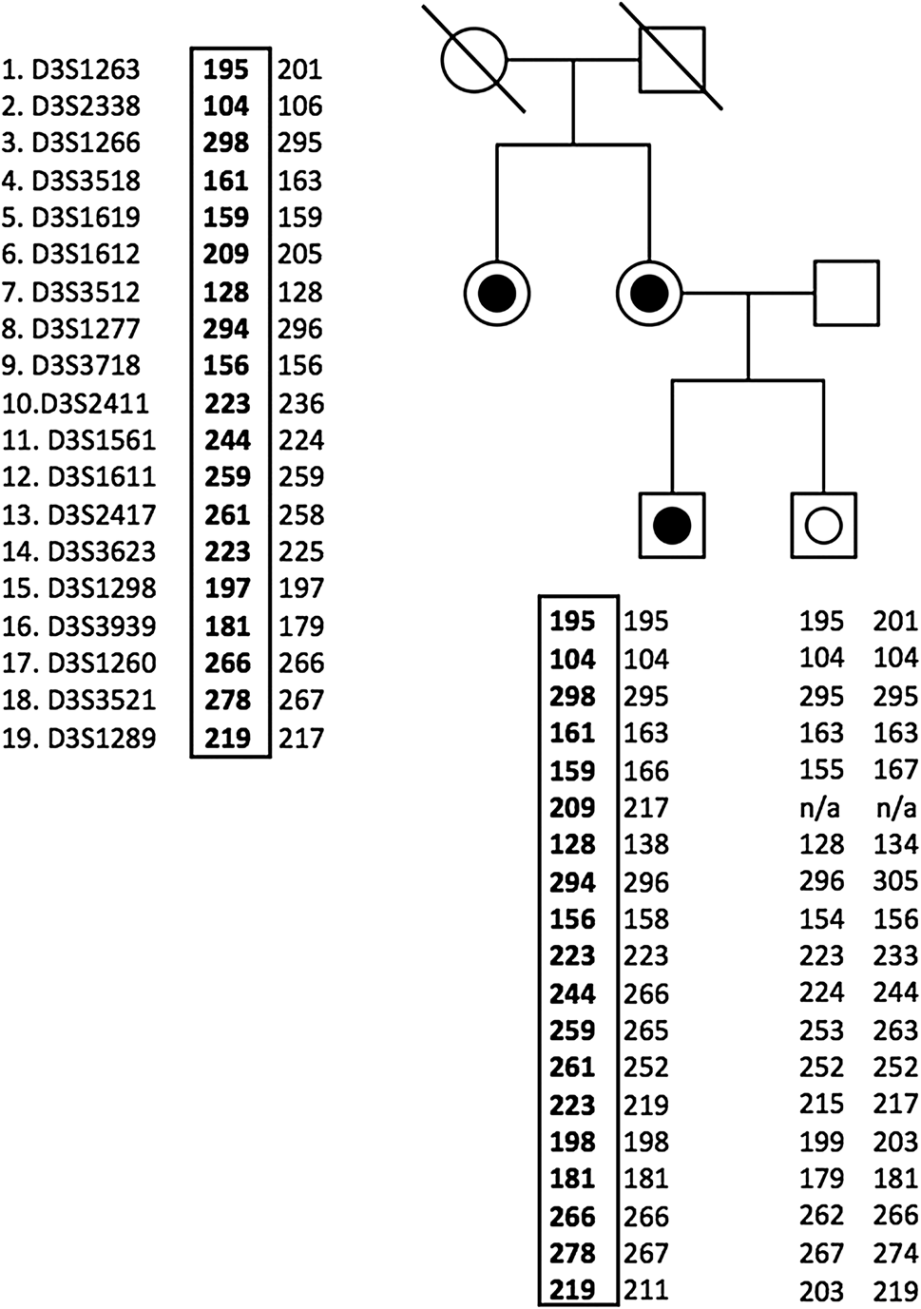
Analyzed markers on chromosome 3p22 in family 1552. The disease associated haplotype is marked with a box and in bold letters. Individual with a black dot indicates this is a verified mutation carrier, while a non filled dot indicates a verified non-mutation carrier

In order to determine the haplotype that carried the mutated allele, haplotype analysis was initially performed in a family with more than three known carriers, spanning over more than two generations. Figure 1 displays the shared haplotype and the relation between the analyzed individuals. Based on data from one individual each from seven unrelated Swedish families (Families 1552, 1894, 19, 765, 1197, 1517, 2143), we found a shared haplotype of about 0.9–2.8 Mb (minimum and maximum distance respectively) within the markers D3S1277 and D3S2417 surrounding *MLH1* (human assembly GRCh37, haplotype depicted in Table 2). We then proceeded to analyze this common haplotype in the Finnish (Family 9), the French (Family 10), and the three German families (Families 11–13) as well as one additional Swedish family (Family F0009520) and discovered that the Finnish family and the additional Swedish family shared a haplotype with the Swedish families, while the German and French families shared alleles at some locations but did not share the Swedish haplotype (Table 2).

**Table 2:**
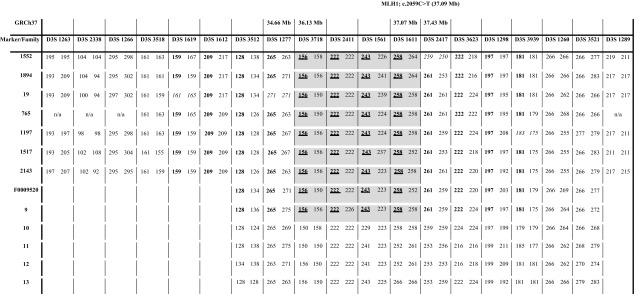
Markers analyzed in eight Swedish families (1552, 1894, 19, 765, 1197, 1517, 2143 and F0009520), one Finnish family (9), one French family (10) and three German families (11–13)

The shared disease associated haplotype in the Finnish and Swedish families is highlighted in gray, with the disease allele in underlined bold letters. Other common alleles are illustrated in bold numbers and positions of potential genetic crossover is shown in italic numbers. Physical positions of markers and the mutation in *MLH1* according to GRCh37 are indicated at the top of the table

## Discussion

LS is an autosomal dominantly inherited cancer syndrome with high penetrance, characterized by primarily early onset colorectal and endometrial cancer. The syndrome is caused by inherited mutations in any of the five MMR genes *MLH1, MSH2, MSH6, EPCAM* or *PMS2*. A number of pathogenic founder mutations have been described in those genes, which are shared by apparently unrelated families that inherited them from a common ancestor. In this study, we have identified a shared haplotype of 0.9–2.9 Mb in eight Swedish families and one Finnish family with Swedish ancestry using 19 microsatellite polymorphic markers surrounding the Lynch syndrome associated mutation *MLH1* c.2059C>T, (p.Arg687Trp). At marker DS31277, family 19 carried the disease allele 271 instead of the common 265 allele (Table 2). Family 1552 shows a recombination event between marker D3S1611 and D3S2417. Since the downstream as well as upstream alleles of these families were consistent with the founder alleles in the common haplotype, this might indicate mutation events rather than a recombination event and thus would implicate that the shared haplotype is larger than 2.9 Mb. Nonetheless, the alleles shared by the Swedish and Finnish family are at a genomic segment of a minimum of 0.9–2.8 Mb.

The earliest verified case in seven of the Swedish families was in the nineteenth century and occurred in individuals from a geographical area in the middle-north of Sweden, except for one family which had ancestors from the very north part of Sweden, as did the Finnish family. Noteworthy, there are two additional Swedish families previously identified with this mutation [5] living in the northern part of Sweden. However, samples from these families were not available for haplotype analysis at the time of this study.

In family number 19, 1197 and 2143, the *MLH1* c.2059C>T mutation does not perfectly segregate with cancer diagnosis (Table 1). Mutations causing Lynch syndrome are indeed characterized by heterogeneity both in penetrance and phenotype, however in these families this segregation pattern is probably due to random events of sporadic cancer, and in some cases due to young age in known mutation carriers. Importantly, the contribution of MMR mutations to Lynch syndrome associated cancer is a function of each patient’s genetic and environmental background, influencing mutation penetrance. Therefore, a more limited genetic variability would be beneficial in the study of cancer risks. This can be offered in members of founder populations such as *MLH1* c.2059C>T, and might aid in more personalized cancer-risk counseling for those patients.

The *MLH1* c.2059C>T mutation occurs independently both in Europe [18–20], Asia [21] and Australia [22, 23], suggesting that globally this is a recurring mutation, while in Sweden it seems to represent a founder mutation which arose in a common ancestor that existed several generations back. Genetic mutations situated in mutational hotspots might actually be prevalent in several populations, but can still display a founder effect in specific populations. This is exemplified by two deletions in *MSH2* detected in Portuguese families that shared disease associated haplotype. On the contrary, in families from Germany, Scotland, England and Argentina carrying the same mutation the haplotype was different [24, 25]. The authors explained this recurrence by a short repeated sequence motif upstream the mutation that created a mutational hotspot in *MSH2*. A similar case is a splice site mutation in *MSH2* that was spread by a founder in Newfoundland, but later turned out to occur in several other populations [26]. This mutation is common and arises repeatedly de novo due to sequence features affecting replication, a mononucleotide tract of adenines, with Newfoundland carriers sharing haplotype as opposed to carriers in Hong Kong, Japan, Italy or England [27].

The genome aggregation database (gnomAD, http://gnomad.broadinstitute.org/) [28] reports a carrier frequency of approximately 1/67,000 in the European population (including the Finnish population) for *MLH1* c.2059C>T. In gnomAD the variant has also been found three times in the south Asian population as well as once in the African population, giving a total average carrier frequency of 1/25,000 combining analyzed populations in this database.

In an isolated, growing population the effect of genetic drift/chance is more pronounced than in a relatively heterogeneous population such as the Swedish. Still, there are examples of founder mutations that occur rarely in such populations apart from *MLH1* c.2059C>T, e.g. the mutation c.589-2A>G that affect splicing in *MLH1*. This mutation was detected in ten American and three Italian families, with the American and Italian families having different haplotypes [12].

In conclusion, we show that *MLH1* c.2059C>T mutation is a Swedish founder mutation with a probable origin in a single founder individual in the north of Sweden, whose descendants have migrated southwards in Sweden as well as across the border to Finland. As common genetic variation (e.g. single nucleotide polymorphisms) might also influence disease risks in MMR mutation carriers, information regarding shared haplotypes among founder mutations carriers is useful for more precise risk estimation in the near future. Phenotypic variation in LS among families carrying the same founder mutations has been reported [29], still we emphasize the importance of clinical characterization of founder mutations and additional epidemiologic studies on LS cohorts carrying founder mutations when striving towards mutation-specific counseling and a possibility to improve clinical care.
